# Predictors for patient satisfaction of a single intra-articular injection of crosslinked hyaluronic acid combined with mannitol (HANOX-M-XL) in patients with temporomandibular joint osteoarthritis. Results of a prospective open-label pilot study (HAPPYMINI-ARTEMIS trial)

**DOI:** 10.1186/s12891-022-05352-3

**Published:** 2022-04-27

**Authors:** Dominique Baron, Hugo Baron, Catherine Baerer, Céline Bodere, Thierry Conrozier

**Affiliations:** 1 Consultation pluridisciplinaire de la douleur, Centre de réadaptation fonctionnelle de Lannion-Trestel, Trévou-Tréguignec, France; 2 Cabinet de chirurgie dentaire, Parc d’activités de Coataner, Douarnenez, France; 3 Faculté d’odontologie, Département des sciences anatomiques, Université de Bretagne Occidentale UBO, Brest, France; 4 Service de rhumatologie, Hôpital Nord Franche-Comté, CS 10499 Trévenans, 90015 Belfort, France

**Keywords:** Osteoarthritis, Temporomandibular joint, Crosslinked, Hyaluronic acid, Intra-articular injection

## Abstract

**Background:**

Chronic pain and functional impairment interfere with the quality of life of subjects suffering from temporomandibular joint (TMJ) disorders. Intra-articular (IA) hyaluronic acid (HA) injections have been shown to alleviate pain and improve mandibular mobility in patients with TMJ osteoarthritis (OA).

**Objectives:**

The primary aim of the study was to identify the prognostic factors of patient satisfaction for a single IA injection of a mannitol-modified crosslinked HA (HANOX-M-XL) in patients with TMJ-OA. The second goal was to obtain clinical data on effectiveness, safety and mandibular mobility throughout a six-month follow-up period.

**Patients and methods:**

This was an observational single-arm prospective trial with a six-month follow-up. Inclusion criteria: patients with TMJ-OA which is not relieved by analgesics and/or non-steroidal-anti-inflammatory drugs and/or orthotics, with radiological evidence of TMJ-OA. All patients received a single IA injection of 1 ml HANOX-M-XL in the target TMJ. The primary endpoint was patient satisfaction on day 180. The main secondary outcome measures were pain variation on a 11-point numeric scale (0–11) between the date of injection and month six, the variation over time of the Maximum Inter-Incisal Opening Distance (MIIOD) and the patient’s assessment of effectiveness. Predictive factors of success or failure were also studied. All adverse events were recorded.

**Results:**

36 subjects (mean age 55.3 years, mean disease duration 98 months), covering a total of 52 injected TMJs, were included. Between baseline and endpoint, the average pain while chewing decreased dramatically from 6.9 ± 1.2 to 2.9 ± 1.3 (*p* < 0.0001) and the MIIOD increased from 29 ± 7 to 35 ± 5 mm (*p* < 0.01). On day 180, all patients were satisfied with the treatment, with 34 patients (94%) rating it as highly effective or effective. Tolerability was good in all but one patient. In the multivariate analysis, patient satisfaction on day 180 was highly correlated with the pain while chewing score, pain on palpation score and the decrease of pain over time (all *p* < 0.0001) but not with MIIOD, gender, age, bruxism, articular noise and symptom duration. Previous viscosupplementation was also related to higher satisfaction (*p* = 0.01).

**Conclusion:**

Despite a long history of pain, most of the patients with symptomatic TMJ-OA benefited from a single injection of HANOX-M-XL, as shown by the sustained (up to 6 months) decrease in pain and improvement in mandibular mobility, with no safety concerns.

**Supplementary Information:**

The online version contains supplementary material available at 10.1186/s12891-022-05352-3.

Temporomandibular disorders are the most common cause of pain of non-dental origin in the orofacial region, affecting 21.5 to 51.8% of adults [1]. Temporomandibular joint (TMJ) internal derangements include disc displacements,

with or without reduction, that are often responsible for joint sounds, pain and discomfort in the TMJ area. Usually, joint displacements are strictly related to the structure and cinematics of the TMJ and masticatory system, although they can be also caused by the peculiar anatomical morphology of the condyle, glenoid fossa and/or articular eminence [2]. Furthermore, age, dentition and condition of masticatory muscle could be important factors in determining or maintaining TMJ dislocations. Disc displacement is an intracapsular dysfunction that may lead in some cases to degenerative changes in the disc and articular surface [3].

TMJ osteoarthritis (OA) is a degenerative condition, often caused by increased load on the joint, consisting of progressive loss of articular cartilage, subchondral sclerosis and abnormal bone formation (osteophytes) leading to joint damage of the mandibular condyle and the glenoid fossa. The main clinical features of TMJ OA are pain while chewing, joint sounds (clicking or crepitus), restricted motion and mouth opening, and more rarely loss of joint function [4]. The clinical management of TMJ OA includes non-invasive, conservative and multidisciplinary therapies. However, evidence of the effectiveness of these therapies is weak and the results are often disappointing. Conservative treatments for TMJ OA include the correction of occlusal abnormalities, physical therapy, isometric exercises, analgesics, non-steroidal anti-inflammatory drugs (NSAIDs) and intra-articular (IA) injections of corticosteroids, hyaluronic acid (HA) and platelet rich plasma (PRP). If first-line therapies fail, more invasive treatments are considered.

A recently published systematic review showed that TMJ OA has a negative effect on quality of life [5], which supports the need for more research on the prevention, diagnosis and treatment of this condition.

Takahashi et al. showed that the concentration of high molecular weight HA in the synovial fluid (SF) of patients with TMJ OA is decreased due to depolymerization by reactive oxygen species (ROS) and the production of HA molecules with a molecular weight lower than normal [6]. These changes reduce the SF’s lubricating properties, contributing to OA progression, as articular cartilage and synovial connective tissue are submitted to increased mechanical stress. These findings suggest that IA injections of a high molecular weight HA may restore the concentration of HA in the SF and help restore TMJ homeostasis and function [7].

As IA HA injections, also known as viscosupplementation (VS), are effective in decreasing pain and improving function in knee OA, it is logical to draw the hypothesis that similar benefits could be achieved in the treatment of TMJ OA [8–13].

Despite several encouraging findings achieved with HA injections in the management of TMJ OA symptoms, the best protocol to be routinely adopted in the clinical setting has not been formally determined. The identification of reliable outcome predictors is the first step that remains to be achieved in order to improve the use of HA products in TMJ OA management and to better identify specific indications and the best patients to be selected for viscosupplementation therapy.

The primary aim of the present study was to look for predictive factors of patient satisfaction with a single IA injection of crosslinked high molecular weight HA combined with mannitol (HANOX-M-XL) in patients suffering from TMJ OA. The second objective was to obtain prospective data on the safety and efficacy of HANOX-M-XL, which has already been proven to be safe and effective in OA of the knee [14], hip [15] and trapezometacarpial joint [16].

## Ethics approval and consent to participate

The study received approval from the *Comité Consultatif sur le Traitement de l’Information en matière de Recherche dans le domaine de la Santé* (CCTIRS, the Consultation Committee on the Processing of Health Sector Research Information) and of the *Commission Nationale de l’Informatique et des Libertés* (CNIL, the National Committee for Information Technology and Civil Liberties). It has been carried out in accordance with Good Clinical Practice and the Declaration of Helsinki. Before enrolment, patients were required to provide informed consent and were free to withdraw at any time for any reason.

## Declarations

The ARTEMIS Study has been registered by the French *Agence Nationale de Sécurité du Médicament* (ANSM, the National Drug Safety Agency) under the name of HAPPYMINI-ARTEMIS trial (EUDRACT No. 2016-A0017744). The study was registered on August 13, 2018 under ClinicalTrials.gov Identifier NCT03627429.

## Patients and methods

The study was a prospective, open-label, single-centre trial with a six-month follow-up.

### Study population

All of the patients were recruited during a multidisciplinary chronic pain consultation at the Lannion-Trestel Hospital Centre, France. Patients with symptomatic TMJ OA that is not sufficiently relieved by usual first-line treatments and who had undergone a radiography showing evidence of OA (joint space narrowing and/or osteophyte), and for whom the indication of viscosupplementation had been approved by the rheumatologist or dental surgeon, could be included in the trial. Patients with other TMJ involvement (internal derangement related to disc displacement, rheumatoid arthritis and other inflammatory TMJ involvement), those who received viscosupplementation in the target joint within the last 6 months, or corticosteroids during the previous 3 months, cannot be included in the trial. Patients with contraindications to viscosupplementation (i.e. hypersensitivity to HA or mannitol) or to the IA injection procedure (infectious disease in progress, infected skin lesions next to the injection area, clotting disease, etc.) and those in whom the treatment cannot be effectively assessed, who did not speak French or who were unable to give their informed consent were not able to be included in the trial.

### Intervention

All patients received a single IA injection of 1 ml of HANOX-M-XL (HappyMini®, LABRHA Laboratory, Lyon, France) in the first TMJ. To reduce the number of IA injections, HA crosslinking processes have been developed. Crosslinking consists of binding HA molecules together using a crosslinking agent (1,4-Butanediol diglycidyl ether -BDDE, vinyl-sulfone, formaldehyde, etc.) with the aim of protecting the HA from enzymatic and ROS degradation and thus increasing its IA persistence and viscosity [17]. The addition of mannitol (3.5%), a powerful antioxidant polyol, has also been shown to significantly decrease the rate of HA degradation [18] and to reduce its onset of action [19]. HANOX-M-XL is a HA viscosupplement specifically designed for small joints, made up of BDDE crosslinked high molecular weight HA (16 mg/ml) combined with 35 mg/ml of mannitol, that delays the in situ degradation of HA. These specificities allow HANOX-M-XL to be injected using a single injection regimen. Injections were performed by one single very experienced physician as per the following procedure.

The patient was positioned in the lateral decubitus position, on the healthy side. After careful disinfection with povidone, an iodinated derivative (Betadine dermique® 10%, Mylan Medical SAS, France) was applied and, if necessary, the pre-auricular zone was shaved, 1 cm below and in front of the tragus, so as to properly identify the condylar process. Then, the patient was asked to open their mouth slowly by about 2 cm. Under fluoroscopic control, a 25 gauge/25 mm needle was introduced in the temporomandibular joint. In the event of synovial effusion, the latter was removed before injecting HA. If no synovial fluid can be removed, 0.1 ml to 0.5 ml of ioxaglic acid, 320 mg iodine/ml (Hexabrix® 320 mg, Laboratoire GUERBET, France) was injected, allowing the visualization of a linear image of about 1 cm, confirming the accurate positioning of the needle. The contrast agent was then removed, and 1 ml of HANOX-M-XL was slowly injected. Again, the patient was asked to open and close their mouth several times. Lastly, a dressing was placed on the injection point.

### Study design

Outpatients referred to multidisciplinary orofacial pain consultations at Lannion Hospital Centre were recruited by a dental surgeon algologist or a rheumatologist if experiencing the following symptoms: unilateral or bilateral TMJ pain, at rest or while chewing, impairment of jaw movements on maximum voluntary effort and/or assisted opening of the jaw. The degenerative origin of TMJ involvement was confirmed using the DC/TMD including the imaging criteria [20]. Diagnosis was based on radiographic evidence of TMJ OA on both dental panoramic X-ray and Computed Tomography (CT) or Magnetic Resonance Imaging (MRI).

Patients with temporo-mandibular disorders from other origins (i.e myalgia, myofascial pain …) were not included in the trial. The In the present study, attempts to identify outcome predictors were focused on features that any healthcare centre can easily record on their clinical record forms (sex, age, pain levels while chewing and on TMJ palpation, pain duration, unilateral or bilateral injection, NSAIDs and analgesic use).

Because the patient’s judgement is the most used outcome measure in daily clinical practice, patient satisfaction was selected as the primary criteria, from which the predictive factors were assessed. The level of patient satisfaction was rated ‘yes’ (very satisfied/satisfied) or ‘no’ (hardly satisfied/not satisfied).

Response to treatment was also evaluated with two other tools: patient assessment of effectiveness (very effective/effective = yes, hardly effective/not effective = no) and a decrease in pain of at least 50% on the numeric scale.

### Course of the study

#### Selection visit

Before enrolment, patients were required to give their informed consent to participate. During the selection visit on day 0 (D0), the following data were collected: demographics (sex, age, height, weight), duration of pain, target TMJ, previous and current treatments for the TMJ OA (NSAIDs, analgesics, specific rehabilitation of the buccal sphere, wearing an occlusal splint, previous IA corticosteroid injection, previous IA–HA injections, etc.). The investigators asked the patients (and their spouse, if any) if they suffered from bruxism at night or if they felt noises and/or crackles during chewing. TMJ pain while chewing and on palpation was assessed using a numeric scale (NS): 0–10 (0 = no pain, 10 = maximum pain). The Maximum Inter-Incisal Opening Distance (MIIOD) was measured with a Vernier calliper in millimetres (mm) as the vertical distance between the maxillary and the mandibular central incisor edges.

At the end of the selection visit, the IA injection of HANOX-M-XL was performed or scheduled, mostly for organizational reasons, within a maximum of 15 days, in order to limit the risk of variation in clinical state between the evaluation and the day of injection.

#### Follow-up visits at three and six months

During the visits in month 3 (D90) and month 6 (D180) following the injection, the following data were collected: TMJ pain while chewing (NS 0–10), TMJ pain on palpation (NS 0–10), MIIOD (mm), patient satisfaction with the treatment (Likert scale comprising four points, where 0 means ‘not satisfied’, 1 ‘hardly satisfied’, 2 ‘satisfied’ and 3 ‘very satisfied’), patient perception of the effectiveness of the treatment (Likert scale comprising four points, from 0, ‘not effective’, to 3 ‘very effective’), patient perception of pain reduction across six categories ([0%], [1–24%], [25–49%], [50–74%], [75–99%], [100%]), and variation in consumption of analgesics or NSAIDs compared to the baseline ([yes/no] and if [yes], the quantification of such on a five-point Likert scale ([1–24%], [25–49%], [50–74%], [75–99%], [100%]). During each assessment visit, all of the adverse events were recorded.

### Statistics

Baseline and six-month follow-up data are provided as a number, percentage or mean value [CI 95%]. We looked for the predictive factors of response or non-response, first in univariate then in multivariate analysis, by integrating all of the factors with a *p*-value < 0.2 in the univariate analysis, as well as possible confounding factors such as patient age, sex, NSAIDs intake and if they wear an occlusal splint.

The main analysis was intended to be carried out in the per protocol (PP) population using only patients for whom all of the data on day 0 and day 180 were available, then confirmed in the intend-to-treat (ITT) population by processing missing data with the Last Observation Carried Forward (LOCF) method. Mann-Whitney or chi-square tests were used, as appropriate, to assess the association of quantitative or qualitative factors with patient satisfaction. The regression coefficients of the multivariate models (ANCOVA and mixed model) were considered significant if they were less than 5%. Statistics were achieved using XLstats software© (Addinsoft, Paris, France).

## Results

Thirty-six patients (27 female, 9 male) were included in the trial. There was no drop-out, so the statistical analysis was performed on the total patient sample. The average age (range) was 55.3 years (range 27 to 72 years). Fifty-two TMJs were injected, since 20 patients suffered from a single TMJ disorder and 16 suffered bilateral TMJ involvement. The mean (range) symptom duration was 98 months (2–480). Most of the patients were taking analgesics and/or NSAIDS and wore an occlusal splint. Detailed characteristics of the patients at baseline are given in Table 1.

**Table 1:**
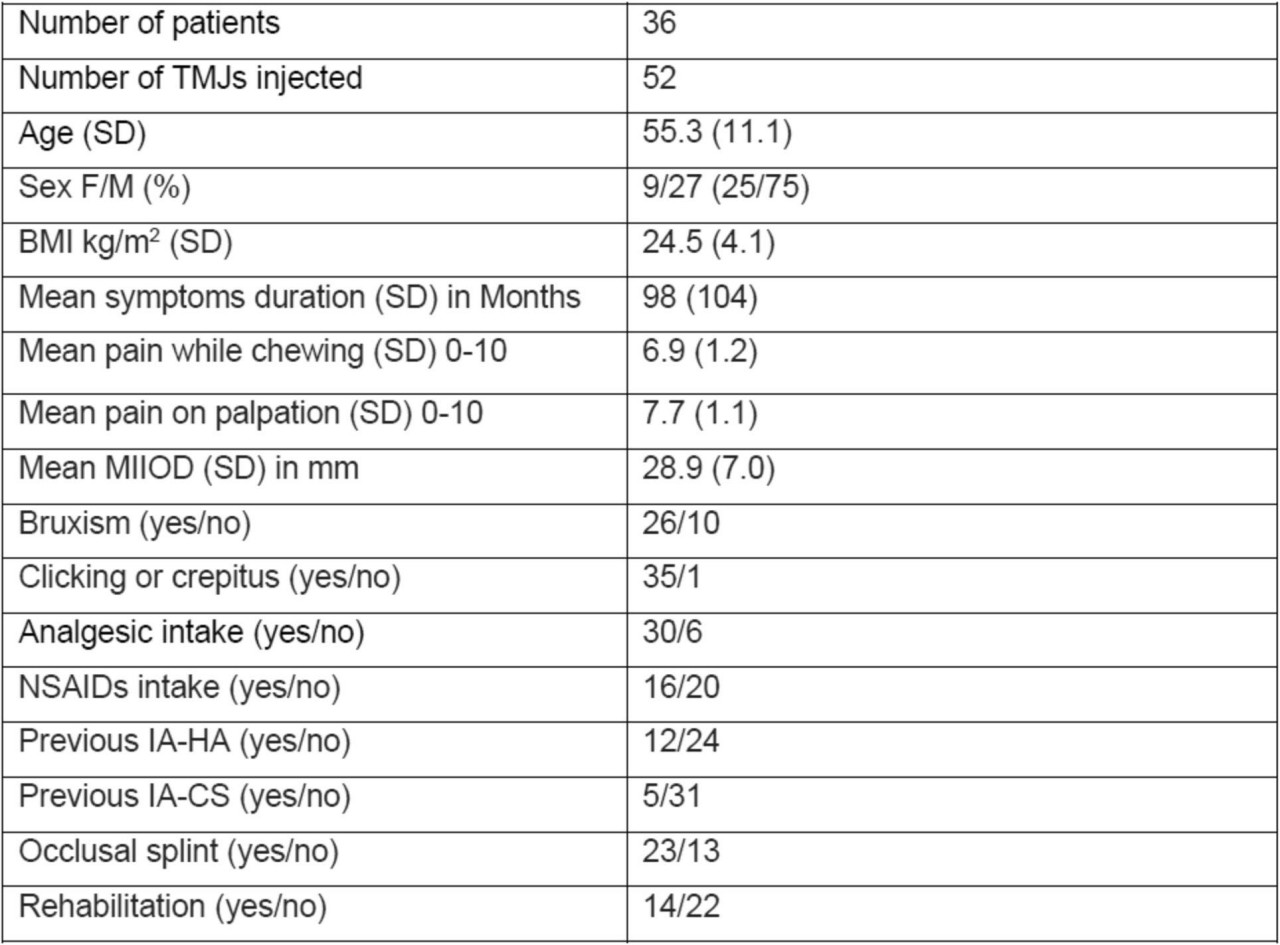
Characteristics of 36 patients with temporomandibular joint osteoarthritis, at baseline

*TMJ* Temporomandibular joint, *SD* Standard deviation, *F/M* Female/male, *BMI* Body mass index, *MIIOD* Maximum inter incisive opening distance, *IA-HA* Intra articular hyaluronic acid, *IA-CS* Intra articular corticosteroid

Ninety days after the injection of HANOX-M-XL, 36% of patients (*N* = 13) were very satisfied, 61% (*N* = 22) were satisfied and 1 subject was hardly satisfied. Pain while chewing decreased significantly from 6.9 (1.2) to 3.4 (1.3) and pain on palpation from 7.7 (1.1) to 3.8 (1.3) (all *p* < 0.0001), The MIOOD increased significantly from 29 (7) mm to 36 (6) mm (=0.005). A decrease in analgesics or NSAIDs consumption was reported by 61.3% of the patients.

**Fig. 1:**
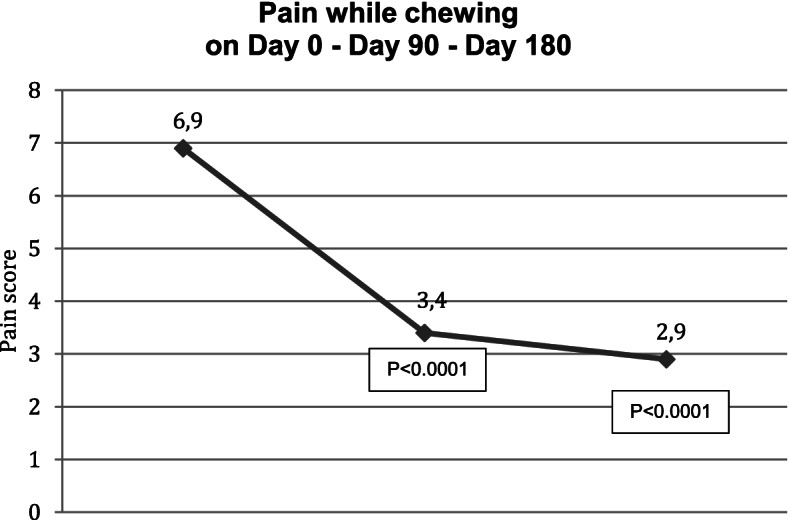
Variation over time of pain while chewing in patients with symptomatic temporomandibular joint osteoarthritis treated with a single intra-articular injection of HANOX-M-XL 1 ml

**Fig. 2:**
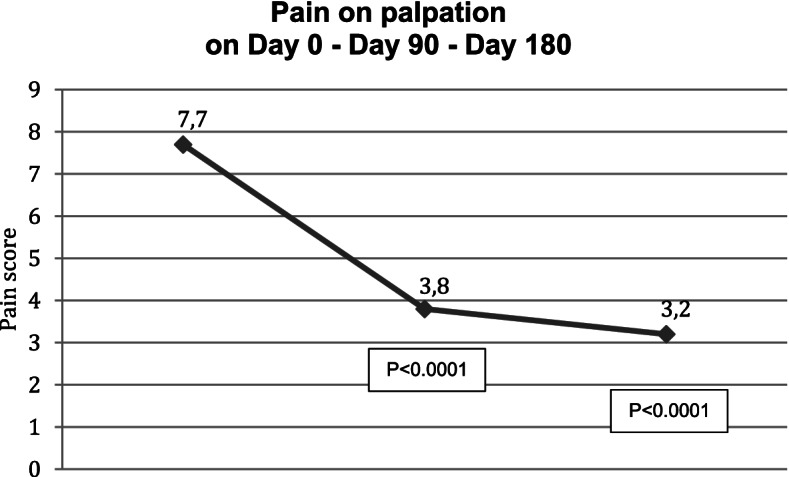
Variation over time of pain on palpation in patients with symptomatic temporomandibular joint osteoarthritis treated with a single intra-articular injection of HANOX-M-XL 1 ml

**Fig. 3:**
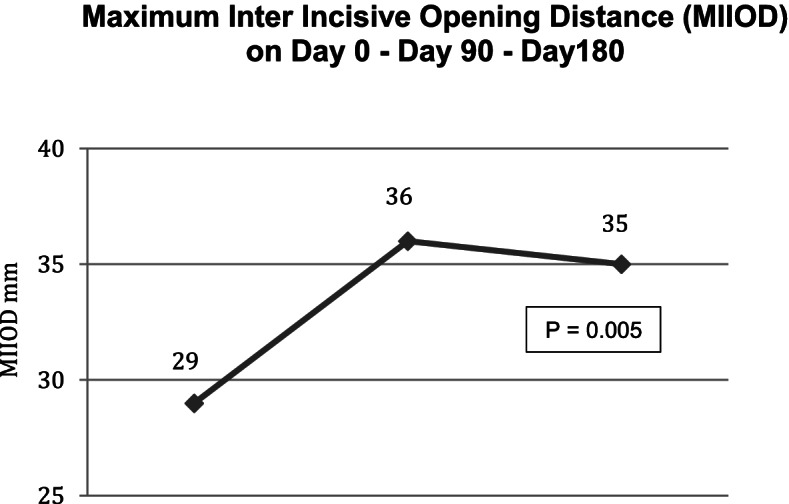
Variation over time of the Maximum Inter Incisive Opening Distance in patients with temporomandibular joint osteoarthritis treated with a single injection of HANOX-M-XL 1 ml

Between D90 and D180, pain continued to decrease, reaching 2.9 (1.3) and 3.2 (1.4) on D180 for pain while chewing and on palpation respectively (p versus baseline < 0.0001). On D180, MIOOD was not significantly different to that measured on D90 (6.0 + 2.0 mm). All patients were satisfied with the treatment (22 satisfied and 14 very satisfied) and 81% of analgesics/NSAIDs users had decreased their drug consumption. The reduction in pain killers was > 50% in half of those patients. No complications or side effects were observed in any patient but one, who experienced local pain during injection due to a technical problem. Outcomes in month three and month six are given in Table 2 and Figs. 1, 2 and 3.

**Table 2:**
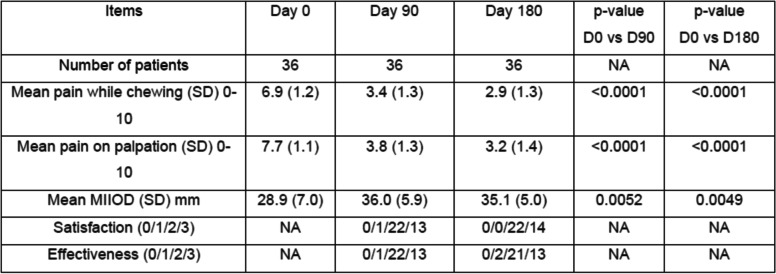
Efficacy outcomes on day 0, day 90 and day 180 in 36 patients with symptomatic temporomandibular joint osteoarthritis treated with a single injection of HANOX-M-XL 1 ml

*SD* Standard deviation, *MIIOD* Maximum inter incisive opening distance

Satisfaction: 1 = not satisfied, 2 = hardly satisfied, 2 = satisfied, 3 = very satisfied

Effectiveness: 1 = not effective, 2 = hardly effective, 2 = effective, 3 = very effective

Among the 36 patients recruited, 18 received unilateral injections and 15 bilateral injections, so we compared the two populations in case the results were different according to the number of joints involved. In the single injection group, all 18 patients were satisfied by month six, versus 14/15 in the bilateral group. There was no inter-group difference in pain level and MIIOD at baseline, or in month three and six. The comparisons made between patients with unilateral and bilateral involvement is provided in Table 3.

**Table 3:**
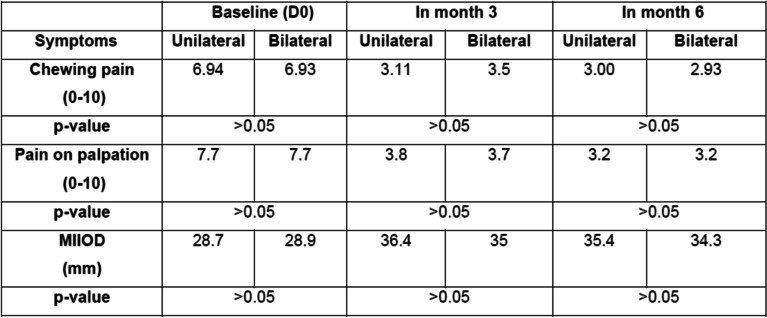
Differences in pain and Maximum Inter Incisive Opening Distance (MIOOD) between patients with unilateral and bilateral temporomandibular joint osteoarthritis treated with a single injection of HANOX-M-XL 1 ml

In the univariate analysis, patient satisfaction on day 180 was highly correlated with satisfaction on day 90 (*p* < 0.001), as well as chewing pain, pain on palpation and the decrease of pain over time (all *p* < 0.0001). Unsurprisingly, patient satisfaction was also highly related to the patient’s assessment of effectiveness on D90 and D180 (*p* < 0.0001). Previous viscosupplementation was significantly related to higher satisfaction (*p* = 0.01), as well as the decrease in pain killer use (*p* = 0.012). There was a trend for higher satisfaction in patients using an occlusal splint (*p* = 0.12) and achieving buccal rehabilitation (*p* = 0.07) and in those with shorter symptom duration (*p* = 0.13), but the difference did not attain statistical significance, likely because the sample size was too small.

Inversely, satisfaction on both D90 and D180 was not correlated with pain on D0 (*p* = 0.63), MIIOD on D0 (*p* = 0.68) and D180 (*p* = 0.46), MIIOD variation over time (*p* = 0.64), gender (*p* = 0.54), age (*p* = 0.19), previous corticosteroid injection (*p* = 0.46), baseline pain killers use (*p* = 0.65), bruxism (*p* = 0.46) or articular noise (*p* = 0.39).

In the multivariate analysis, no prognostic factors of satisfaction were identified.

## Discussion

Because IA HA injections have previously been demonstrated to be effective for relieving durable pain in patients suffering from TMJ OA, the present study was not designed for assessing the effectiveness of viscosupplementation, but specifically to look for prognostic factors of patient satisfaction. Patient satisfaction is a growing concern and plays a crucial role in the healthcare system, the patient’s opinion being increasingly used for assessing treatments effectiveness. Predicting patient satisfaction/dissatisfaction may help practitioners make decisions about viscosupplementation and therefore may optimize the rate of treatment success.

HANOX-M-XL is a second generation viscosupplement [21] combining crosslinked HA and mannitol and allowing, because of its long-lasting IA residence time, a single injection regimen, which is much more comfortable for patients than multiple weekly injections. This may influence patient satisfaction, especially in those who have been treated previously with a multiple injection regimen.

Unfortunately, our study failed to identify predictive factors of patient satisfaction, likely because the rate of satisfaction was extremely high, since all patients remained satisfied with the therapy 6 months after a single IA injection. There was a trend for better results in patients with shorter symptom duration and in those who had previously received a HA injection. It is interesting to underline that a long history of TMJ pain (mean disease duration 98 months), advanced age, a smaller MIIOD and a high level of baseline pain did not significantly reduce the rate of success of the HANOX-M-XL injection. In our study, we did not find any differences in outcome in patients treated for unilateral or bilateral TMJ OA, unlike Guarda-Nardini et al. [14], who found that patients with unilateral TMJ OA benefited more from IA HA injections than those with bilateral involvement. In their study, 22 out of 27 subjects who received treatment only in a single joint had a positive outcome (81.4%) compared to 36 out of 63 subjects with bilateral OA (57.1%).

However, in the absence of a control group, the present pilot study does not allow to formally assert that HANOX-M-XL acts better than a placebo. Although this study was open-label, and not intended to prove the effectiveness of HANOX-M-XL injections for TMJ OA, it is interesting to note that all of the enrolled patients benefited from the treatment, as shown by the percentage of satisfied patients, that reached 100% by month 6. A placebo effect cannot be formally excluded, at least in some patients. However, the percentage of satisfied patients exceeded the expected proportion of placebo responders [22–24]. Furthermore, placebo response is generally mild to moderate whereas, in the present study, 80% of patients exhibited a decrease in pain of more than 50%. Among the other strengths of this study, the fact that the injections were all carried out by a single experienced rheumatologist, using the same procedure, must be mentioned. Safety was good in all patients, the only reported adverse event being pain at the time of injection, which was due to the injection procedure and not to the injected product. Contrary to what is mentioned regarding other joints [18–20], none of our patients reported pain within a few hours or a few days after injection.

However, our study suffers from several limitations. The sample size was small, which likely explains the lack of statistically significant correlations between patient satisfaction and symptom duration, the use of an occlusal splint and buccal rehabilitation. The fact that IA injections were performed without the help of imaging guidance can be considered an important limitation. However, the high rate of responders during months three and six strongly suggests that the TMJs were injected with accuracy. Lastly, we did not collect precise data on the radiological features and therefore cannot explore the relationship between treatment effectiveness and the anatomical severity of OA. Anyway our study confirms the positive data on the effect of HA in TMJ pathologies obtained using different protocols. Kopp et al. first reported in 1985 and 1987 the short- and long-term therapeutic outcomes of HA injections after arthrocentesis of the TMJ, and compared their effects with that of IA injections of corticosteroids [8, 9]. The authors concluded that both drugs were useful for the treatment of TMJ OA, demonstrating good results in the long term. Many studies support the usefulness of IA HA injections in a large percentage of patients with degenerative TMJ disorders. The most common dosing regimen for TMJ VS is five weekly injections preceded by arthrocentesis, although a meta-analysis concludes that washing the TMJ is effective and does not require the addition of another drug [10]. Guarda-Nardini et al. [11] compared the effectiveness of two treatment protocols comprising five weekly arthrocentesis sessions on the TMJ followed by injections of a low or a medium molecular weight HA in patients with TMJ OA-related pain persisting for more than 6 months. Three months after treatment, similar positive effectiveness was shown for the two treatment protocols. Most of the other published data on TMJ OA viscosupplementation come from trials using the same regimen of five weekly injections of low molecular weight HA [12]. Other protocols providing two injections or even a single injection [13] of a higher molecular weight HA also saw positive outcomes.

In conclusion, despite no response predictors being formally identified, this open-label trial gave relevant information on the use of HANOX-M-XL in patients suffering from symptomatic TMJ OA. Six months after a single 1 ml injection in the TMJ, all patients experienced a decrease in pain and an improvement of jaw mobility. All were satisfied with the treatment and 39% said they were highly satisfied with the therapy despite a long history of pain. A single IA injection of HANOX-M-XL might be a new therapeutic option to relieve pain and improve function in patients suffering from TMJ pain of degenerative origin, with no safety concerns. Further controlled studies with larger sample sizes are needed to confirm these promising results.

## Supplementary Information

**Additional file 1.**

## Data Availability

The datasets used and analysed during the current study available on reasonable request from the trial promoter: Laboratoire de Rhumatologie Appliquée, 19 place Tolozan, 69001, Lyon, France (info@labrha.com).
