# Supplementary material for: Predictors for patient satisfaction of a single intra-articular injection of crosslinked hyaluronic acid combined with mannitol (HANOX-M-XL) in patients with temporomandibular joint osteoarthritis. Results of a prospective open-label pilot study (HAPPYMINI-ARTEMIS trial)
Source: BMC Musculoskelet Disord. 2022 Apr 27;23:392. doi: 10.1186/s12891-022-05352-3 (PMC9044650; doi:10.1186/s12891-022-05352-3)
Supplement: Supplementary file 1 — Additional file 1. [file 12891_2022_5352_MOESM1_ESM.pdf]

Observational epidemiological study

EUDRACT N° 2016-A00177-44

ARTEMIS

Treatment of Temporo-Mandibular Arthrosis by intra-articular Injection of hyaluronic acid.

Study of predictive factors of patient satisfaction, 6 months after a single injection of HAPPYMINI®.

PROMOTER : Laboratoire LABRHA, 19 place Tolozan, F-69001, Lyon, France

version 4 of 31/03/2016

1-Proof of research:

Osteoarthritis of the temporomandibular joint (TMJ) is a frequent cause of arthromyalgia of the face grouped under the acronym DAM (Dysfunction of the Manducator Apparatus). It can be expressed by a wide range of symptoms dominated by pain in the TMJs, associated or not with limitations in mouth opening, or even by blockages, joint noises and other signs such as bruxism, otalgia or tinnitus. These pains can be accompanied by functional disorders such as a limitation of the mouth opening, evaluated by the inter-incisor distance (IDI), sometimes linked to a dislocation of the disc (or meniscus) which is interposed between the condylar process of the mandible and the glenoid cavity of the temporal bone. The management of this pain is essentially based on empirical means, prescribed out of habit and without any real evaluation (analgesics, NSAIDs, muscle relaxants) as well as on the wearing of occlusal splints and/or occlusal equilibration by adding composite resins. In the event of failure of first-line treatments, the treatment is based on corticosteroid infiltrations, the effectiveness of which is transient, or on the injection of hyaluronic acid (HA) also called viscosupplementation.

A meta-analysis including 19 studies showed that in DAM in general (12 with disc problems and 7 with inflammatory flare-ups of degenerative arthropathy) (1) HA injections provide a greater reduction in pain than placebo (saline solution) in all cases where it was compared to it, and overall comparable to corticosteroid infiltrations. In another meta-analysis that included 18 studies on the use of HA in TMJ osteoarthritis, Escoda-Francolí et al (2) reached similar conclusions and proposed a "Strong" strength of recommendation regarding the indication of viscosupplementation in degenerative TMJ disorders (osteoarthritis and meniscopathy). Finally, the authors of the Cochrane review (3) conclude that HA injections provide a long-term improvement in the symptoms of TMJ osteoarthritis, that their beneficial effects are equivalent to those of glucocorticoids, and that one can optimize their results by combining them with arthrocentesis.

The objective of this research is therefore not to demonstrate the effectiveness of viscosupplementation in painful degenerative pathologies of TMJ **but to seek clinical factors predicting the success or failure of treatment** such as clinical severity before the injection, the presence of a limitation of the mouth opening or the duration of the symptoms.

For this we place ourselves **in the conditions of daily practice, in patients with symptomatic temporomandibular osteoarthritis with no other selection criteria** than the opinion of the investigator concerning the merits of viscosupplementation and the agreement of the patient participating in the research**. It in no way modifies the patient's therapeutic management and imposes no other constraint on him than filling in simple questionnaires commonly used in daily practice, in the monitoring of this pathology.**

2- Population concerned:

Forty patients with temporomandibular osteoarthritis confirmed radiologically by a pantogram dating less than 12 months and justifying, in the opinion of the rheumatologist and/or the dental surgeon, viscosupplementation.

Inclusion criteria:

Those in **daily clinical practice**, i.e. all ambulatory patients over 18 years of age, of both sexes, whose clinical diagnosis of temporomandibular osteoarthritis is confirmed by a dental panoramic radiograph less than 12 months old, and for which the indication for viscosupplementation has been selected by the medical specialist, rheumatologist or dental surgeon, on the basis of the usual clinical criteria (pain, discomfort when chewing, insufficient efficacy or poor tolerance of analgesics and/or anti-inflammatories).

Exclusion criteria:

- Patients with a contraindication to viscosupplementation with HAppymini® hypersensitivity to hyaluronic acid or mannitol) or to

intra-articular injection procedure (current general infectious disease, infected skin lesions near the injection site, dental infection).

- Patients in whom the evaluation of the effectiveness of the treatment could not be carried out reliably:

  - Patients who received a viscosupplement in the target joint during the previous 6 months,
  - Patients who have received an intra-articular injection of corticosteroids in the target joint during the previous 3 months,
  - Patients undergoing planned surgery within 3 months of injection that may interfere with follow-up or evaluation,
  - Patients who do not speak French or who are unable to give their consent personally.

3- Course of the study

Single-center study carried out within the framework of the multidisciplinary consultation for chronic stubborn pain at the Lannion-Trestel Hospital Center, 22660 Trévou-Tréguignec. The recruitment of patients and their follow-up will be carried out by the dental surgeon specializing in occlusology, and/or the rheumatologist of the multidisciplinary consultation for oro-facial pain.

3-1. During the selection visit (D0), the following elements will be collected:

- Demographics (gender, age, height, weight)
- Length of pain
- The target side

- Previous treatments for the pathology evaluated (NSAIDs, analgesics, muscle relaxants, infiltration of corticosteroids into the painful TMJ, injections of hyaluronic acid into the painful TMJ, specific rehabilitation of the oral sphere, wearing an occlusal splint)
- The search for bruxism, noises and / or crackles during chewing,
- TMJ pain on chewing measured on an 11-point numerical scale, "0" meaning "no pain" and "10" meaning "intolerable pain" (Likert 0-10),
- TMJ pain on palpation (Likert 0-10),
- The inter-incisor distance (IID) at the maximum opening of the mouth, measured in millimeters using a caliper.

3-2. During the visits at 3 (D90) and 6 months after the injection (D180) the following elements will be collected:

- TMJ pain when chewing (Likert 0-10),
- TMJ pain on palpation (Likert 0-10),
- IID at maximum mouth opening (mm)
- Patient satisfaction with the treatment (4-point Likert where 0 means “Not satisfied” and 3 means “Very satisfied”)
- The patient's perception of the effectiveness of the treatment (4-point Likert where 0 means "Not effective" and 3 means "Very effective")
- The patient's perception of the reduction in pain in 6 categories: [0%]; [1-24%], [25-49%], [50-74%], [75%-99%], [100%]
- The change in his consumption of analgesics or anti-inflammatories compared to D0 ([YES/NO] and if [YES] quantification on a 5-point Likert scale ([1-24%], [25- 49%], [50-74%], [75%-99%], [100%])
- Any adverse effects during the D90 visit (to ensure quality monitoring of the treatment)

4- Method of investigation retained

The study is observational, prospective, open, monocentric carried out under the conditions of daily practice, part of the multidisciplinary consultation for chronic stubborn pain at the Lannion-Trestel Hospital Center.

4-1. During the initial visit, the investigating doctor :

- gives the patient the information note, answers their questions and obtains their oral consent,
- verifies that the inclusion and exclusion criteria are respected,
- completes, in the data collection notebook assigned to the patient, according to the methodology developed in § 3-1,
- fills in the section "treatments for temporomandibular osteoarthritis", all treatments being authorized according to the rules of an observational study, provided they are collected,
- checks that the dental panoramic X-ray is less than 12 months old and asks for another one if necessary, as recommended before any intra-articular injection into the ATM.
- schedules or performs the intra-articular injection of HAppymini® hyaluronic acid himself. The injection may be performed the same day or for organizational reasons within a maximum period of 15 days, in order to limit the risk of variation in the clinical condition between the assessment and the date of the injection.

4-2. During the intermediate visit on D90 (+ or - 14 days) and the final visit on D180 the investigator:

- postpones to the end of the CRF any changes in concomitant treatments for TMJ osteoarthritis,

- reports any adverse events that have occurred since the day of the injection, and will specify whether or not they are attributable to the treatment or the procedure,
- completes the CRF according to the methodology developed in § 3-2.

5- Treatment and injection technique

5-1. The treatment

It will consist of 1 single intra-articular injection into the painful temporomandibular joint of 1ml of a viscosupplement marketed for this indication (HAppymini®, Laboratoire de Rheumatologie Appliquée, Labrha, Lyon). HAppymini® is a sterile, pyrogen-free and isotonic viscoelastic solution of cross-linked sodium hyaluronate in a buffered physiological solution. The sodium hyaluronate contained in this injectable medical device for intra-articular use is obtained by bacterial fermentation and is present at a concentration of 1.6%. It also contains mannitol at a concentration of 3.5% and comes in the form of a 1 ml glass syringe containing 1 ml of solution. The syringe is presented in an individual sterilized protective case. Packaging available: box of 1 syringe, bearing the indications for use and a leaflet, which must be stored between +2°C and +25°C for 30 months (MD technical sheet and CE marking in appendix 4).

The injector may be the investigator himself, or another doctor he has designated.

5-2. The injection technique will be that described in the book "Gestures in rheumatology" (4) and carried out according to the usual recommendations for any intra-articular injection, according to a perfectly aseptic administration technique (cutaneous asepsis, wearing of surgical gloves).

5-3. After the injection, patients can go home, observing the usual precautions that follow any intra-articular injection. The investigator is

free of the type of joint saving it will provide (absence, relative, wearing a splint) and its duration, provided it is collected in the CRF.

6- Origin, nature and mode of circulation of the personal data collected

The medical data collected for each patient included will be entered into the CRFs provided by the sponsor. A CRF, indicating the initial of the first and last name of the patient, as well as the serial number of entry into the study, is assigned to each patient. The investigating doctor is responsible for filling in all the required information.

The promoter's CRA will keep the calendar of inclusions up to date, will check with the investigators that the injections have indeed been carried out, and will collect the CRFs once the final visit has been carried out. At each monitoring visit, the sponsor's CRA will retrieve the original sheets of the CRFs already completed and bring them to the sponsor's premises where they will be archived, according to the order number of entry into the study.

At the end of the study, all the data recorded in the CRFs will be entered into an Excel® database. The input work will be carried out within the Labrha research unit. Before the database is frozen, any questions ("queries") will be sent, by the CRA monitor, to the investigators who will have to answer them on the sheets provided for this purpose, by sending them back to the sponsor in a T envelope delivered to investigators. In the event of data not being available, the mention ND will be assigned to the missing variable.

6-1. Data Privacy:

The collection and processing of data by the investigator or the members of his team is carried out with respect for the anonymity of the patients, and the medical information obtained for each patient during this study is considered confidential; any disclosure to a third party is prohibited.

The promoter's CRAs and any person authorized by the promoter must have access to the source documents in order to verify the accuracy of the data entered in the observation book and the application of the regulatory requirements.
The promoter guarantees the respect of the confidentiality of the data of the participants in the study in accordance with the European directives and the French law "Informatique et Libertés".

Patients will be informed of this procedure and of their right to access and rectify data concerning them. During the presentation of the results of the study, the identity of the participating patients will remain confidential.

6-2. Use of information and publication

All information concerning the study is confidential and remains the property of the promoter. The details will only be disclosed to persons involved in the approval or conduct of the study, these persons are bound by professional secrecy in the same way as the investigators, in accordance with article R. 5120 of the CSP.

The investigator can only use this information within the framework of this study and can only use the data concerning the patients if the sponsor gives the authorization. For this, the investigator must present, in detail, the conditions of use of the data, their archiving, and will be in this context, responsible for the database thus created.

At the end of the study, after validation by the coordinator, the study data will be submitted for presentation at scientific congresses (French Society of Rheumatology and one or more international congresses of rheumatology and/or stomatology) and submitted for publication in a peer-reviewed journal chosen jointly by the principal investigator, coordinator and sponsor. In the absence of agreement between the parties, it is up to the promoter to choose the journal to which the manuscript will be submitted.

6-3. Data Archiving

In order to guarantee the protection of patients and the validity of the data from a regulatory point of view, these will be kept for a period of 15 years. No document (paper or electronic) of the study should be destroyed without the prior written consent of the sponsor.

7- Duration of the study

This observational study requires a follow-up period of 6 months. The expected start date of recruitment is 01/04/2016. Recruitment will end when 44 patients have been included, in order to obtain 40 complete files, taking into account a percentage of trial dropouts and loss of follow-up of 10%. Recruitment time is estimated at 6 months. The total planned duration of the study is therefore 12 months.

8- Data analysis method

8-1. The analysis of this observational study will be purely descriptive.

The demographic, clinical and radiographic qualitative variables will be described by their number, percentage and missing data by response modality and the quantitative criteria by their number, average, standard deviation. In the case of quantitative criteria with asymmetrical behavior, they will be presented with their median and the interquartile range (25th percentile - 75th percentile).

8-2. The endpoint is the response to treatment.

The aim of the study being the search **for predictive factors of response to treatment,** it is not relevant to determine a primary endpoint. We will study the possible correlation between the response to the treatment and the level of pain on D0, the duration of the symptoms, the presence of a limitation of the mouth opening, and the existence of radiological lesions of osteoarthritis on the panoramic dental picture.

The response to the treatment will therefore be estimated by 3 complementary methods and for each of them the predictive factors of response (YES/NO) will be studied. Each patient will be classified into a treatment response category: YES or NO depending on:

1. The patient's assessment of the effectiveness (effective and very effective = YES, not or only slightly effective = NO),

2. Reduction in pain of at least 3 points or at least 50% on the Likert scale.

3. The degree of patient satisfaction (Very satisfied and fairly satisfied = YES, little or not satisfied = NO),

We will look for predictive factors of response or non-response, first in univariate analysis then in multivariate analysis by integrating all the factors with a P<0.1 in the univariate analysis, as well as possible confounding factors such as age, gender, taking NSAIDs, wearing a tray.

The main analysis will be carried out on the per-protocol population including only patients for whom all the data at D0 and D180 are available, then confirmed by ITT. For patients who do not have D180 data, the latest data available (D90) will be used (LOCF). The regression coefficients of the multivariate models (ANCOVA and mixed model) will be considered significant if the degrees of significance are less than 5%. Statistical analyzes will be performed with XLStat software.

8-3. Justification of the number of subjects

The study being purely exploratory, the number of subjects required is not possible to determine a priori. A number of 40 patients was therefore chosen by the study's scientific committee, as likely to provide sufficient information to allow hypotheses to be put forward on the question posed while allowing reasonable feasibility.

9- Modalities for stopping the study

9-1. Early termination of the study by a patient

A patient who has agreed to participate in the study may at any time decide to stop participating without any obligation to provide explanations.

9-2. Early termination of the study by the investigator in the interest of a patient

The investigator can at any time decide to stop the study in the interest of a patient.

9-3. Early termination at the promoter's instigation

The sponsor reserves the right to interrupt the study at any time for medical or administrative reasons, if possible after consultation with the coordinating investigator, the study committees and the competent administrative authorities. In this event, an information letter will also be sent to the investigators. The reasons for the stop should be documented there.

10-Vigilance and management of adverse events

As this study is observational and falls within current practice, it is not mandatory to include a section for collecting adverse events.

Nevertheless, the scientific committee and the sponsor wanted a collection as careful as in the interventional studies to be carried out.

10-1 Collection of adverse events by the investigators

- Collection of non-serious adverse events: Investigators must note all adverse events, even non-serious, on the adverse event form in the observation notebook.
- Reporting of serious adverse events: In the event of a serious adverse event, the investigator has the obligation to report it without delay to the

promoter who immediately sends it to the distributor (Laboratoire Labrha) who himself informs the manufacturer of the medical device within 24 hours (Laboratoire Vivacy, Archamps, France), according to the internal procedures of the 2 companies:

- on a specific form attached to the observation book: "Serious Adverse Event Declaration Form",
- form to be sent by email within 24 hours after learning of the event, to the Proponent's Materiovigilance Manager at the following address: thierry.conrozier@labrha.com

If this is not possible, the information must be communicated by telephone within the same timeframe to the Vigilance Manager on 06 86 91 92 18.

The causal link with the medical device and/or its administration procedure must be suggested by the investigator (none, possible, probable, proven).

10-2. Immediate declaration to the National Agency for the Safety of Medicines and Health Products (ANSM)

The sponsor shall immediately report to the competent authority the occurrence of any serious adverse event, serious adverse effect related to the use of the device and/or unexpected adverse effect related to the use of the device that an investigator reports to him. He also informs the other investigators participating in the study.

All information relating to a serious adverse event is integrated into the promoter's vigilance database. A summary is drawn up by the sponsor's pharmacovigilance unit in the form of CIOMS I.

The following main elements are identified on CIOMS I:

- a unique biomedical research identifier (EudraCT number),

- a coding of the event(s) considered serious, expected and unexpected, according to the dictionary of adverse effects terminology (MedDRA),
- imputability according to the investigator and imputability according to the sponsor.

The sponsor declares to the ANSM:

- All unexpected serious adverse effects likely to be due to the DM,
- All serious adverse events likely to be linked to the procedure for implementing the MD,
- Any safety data or new fact that could significantly modify the assessment of the benefit/risk ratio of the MD.

Usually do not require immediate reporting:

- Non-serious side effects,
- Adverse events considered unrelated to the MD or to the procedure for implementing the MD.

As defined in article R.1123-48 of the Public Health Code relating to vigilance in the context of biomedical research, any suspicion of an unexpected serious adverse effect and any serious adverse event that may be linked to the act of implementation of the medical device must be sent to the ANSM Medical Devices Clinical Monitoring and Evaluation unit.

10-3. Follow-up of adverse events

Any adverse event will be monitored by the investigator until return to the initial state or recovery or, failing that, until stabilization of the state; the possible sequelae for the patient will then be mentioned. For serious events, the investigator must endeavor to obtain all the elements necessary for the documentation and clinical evaluation of the observation. For serious events, if additional information is available to assess the case (evolution of the case,

additional examinations, hospital report, etc.), they must be sent by email by the investigator within 24 hours of becoming aware of the event, to the materiovigilance manager at the following address: thierry.conrozier@labrha. com or by telephone to the scientific manager of the study on 06 86 91 92 18.

10-4. List of expected adverse events

- Adverse events related to the injected viscosupplement: The most common (2 to 10%) is transient pain in the treated joint, lasting a few hours to a few days after the injection. More rarely, a painful effusion can be observed (<5%). However, these reactions generally disappear after a few days without any treatment. During its operation, no general reaction was observed with the DM used.

- Adverse events related to the practice of injection: During or after intra-articular injection, transient pain or hematoma at the puncture site may appear. The risk of joint infection is estimated between 1/20,000 and 1/50,000 injections.

11- Ethical aspects and administrative procedures

11-1. Regulations

In accordance with the Public Health Code in its articles L.1121-1, L.1123-8, R.1123-26, Law No. 2004-806 of August 9, 2004, and Chapter IX of Law No. 78-17 of January 6, 1978 relating to data processing, files and freedoms, this study must be registered with the ANSM (National Agency for Medicines and Health Products). The study is not subject to an authorization request from a CPP (Committee for the Protection of Persons). A request for authorization is made to the Advisory Committee on the processing of information in the field of health research (CCTIRS), and the National Commission for Computing and Liberties (CNIL). The contract binding each investigator to the sponsor will be submitted to the National Council of the Order of Physicians (CNOM)

This study will be conducted in accordance with Good Clinical Practices (Decision of November 24, 2006) and the Declaration of Helsinki (Ethical principles applicable to medical research on human subjects, Seoul 2008) and will be registered on the site: https:// clinicaltrials.gov

11-2. Study Committee

The study was written by Dr Dominique Baron, rheumatologist at the Lannion hospital center in Trévou-Tréguignec and by Dr Thierry Conrozier, rheumatologist at the Nord Franche-Comté hospital center in Belfort who also coordinates the research and who can therefore, if necessary where appropriate, propose any substantial modification to be made to the research. He will also be consulted during the validation of the data before the freezing of the study database, the report of which will be written by Dr Baron.

11-3. Amendments for substantial modification

Any changes to the study occurring after protocol approval should be documented as a protocol amendment and submitted to the appropriate authorities if a substantial change to the study protocol has a significant impact on any aspect of the research, including the protection of persons, the conditions of validity of the research, the quality and safety of the products used, the methods of conducting the study.

11-4. Audits and inspections

The sponsor may organize audits of the study by checking the procedures as well as the quality, authenticity and compliance with the source data of the information obtained. Direct access to source documents is necessary to be able to carry out these audits. Similarly, an administrative inspection by an inspector delegated by the competent health authorities is possible: acceptance of the protocol by the investigator also includes acceptance of the principle of this inspection of the study site.

References

1. Manfredini D, Piccotti F, Guarda-Nardini L. Hyaluronic acid in the treatment of TMJ disorders: a systematic review of the literature. Cranio. 2010 Jul;28(3):166-76.

2. Escoda-Francolí J, Vázquez-Delgado E, Gay-Escoda C. Scientific evidence on the usefulness of intraarticular hyaluronic acid injection in the management of temporomandibular dysfunction. Med Oral Patol Oral Cir Bucal. 2010 Jul 1;15(4): 644-8.

3. Shi Z, Guo C, Awad M. Hyaluronate for temporomandibular joint disorders. Cochrane Database Syst Rev. 2003;1:CD002970.

4. Baron D. Infiltration de l’articulation temporo-mandibulaire. In: Baron D. Les gestes en rhumatologie. 2nde édition, Sauramps Médical;2009, 789 pages.
